# Case Report: Aicardi-Goutières Syndrome Type 6 and Dyschromatosis Symmetrica Hereditaria With Congenital Heart Disease and Mitral Valve Calcification – Phenotypic Variants Caused by Adenosine Deaminase Acting on the RNA 1 Gene Homozygous Mutations

**DOI:** 10.3389/fped.2022.852903

**Published:** 2022-06-27

**Authors:** Lingjuan Liu, Lu Zhang, Peng Huang, Jie Xiong, Yangyang Xiao, Cheng Wang, Dingan Mao, Liqun Liu

**Affiliations:** ^1^Department of Pediatrics, The Second Xiangya Hospital of Central South University, Changsha, China; ^2^Department of Pediatric Neurology, Children’s Medical Center, The Second Xiangya Hospital of Central South University, Changsha, China

**Keywords:** AGS 6, DSH, ADAR1 homozygous mutations, PDA, VSD, mitral valve calcification

## Abstract

Dyschromatosis symmetrica hereditaria (DSH), characterized by a mixture of hyper- and hypopigmented macules on the skin, is a rare pigmentary dermatosis of autosomal dominant inheritance. The pathogenic gene is adenosine deaminase acting on the RNA 1 gene (ADAR1), mutations in this gene also lead to Aicardi-Goutières syndrome type 6 (AGS 6), a rare hereditary encephalopathy with isolated spastic paraplegia. The pathomechanism of the ADAR1 gene mutations inducing DSH has not been clarified yet. We report the first case of DSH combined with AGS caused by the homozygous mutation of the ADAR1 gene in China (c.1622T > A) and reviewed the relevant literature. AGS 6 could occur in both men and women, and start in infancy. The main characteristics are growth retardation, skin depigmentation, intracranial calcification, and cerebral white matter lesions. In the current paper, the proband also had patent ductus arteriosus (PDA), ventricular septal defect (VSD), and mitral valve calcification, which are new symptoms that have not been reported in other cases. Additionally, we also aim to discuss the possible molecular mechanisms underlying the clinical heterogeneity caused by ADAR1 gene mutations.

## Introduction

Dyschromatosis symmetrica hereditaria (DSH) shows the pattern of autosomal dominant inheritance. It is characterized by the freckle-like pigmentation or hypopigmentation spots on the backs of hands and feet, presenting a reticulate pattern (1). In infancy, some patients have small freckle-like pigmented macules on their necks and faces that will last a lifetime (2). In 1910, DSH was first described by Toyama, and most of the studies have been widely reported in Chinese and Japanese individuals (3). It has been clarified that heterozygous mutations of the adenosine deaminase acting on the RNA 1 (ADAR1 or DSRAD) gene caused DSH in four Japanese families (4). Pestal et al. reported 26 novel mutations in the ADAR1 gene in DSH families. Subsequently, other novel mutations have been verified in Chinese patients with DSH (5). Up to now, more than 130 different ADAR1 mutations have already been documented in individuals with DSH (6).

Adenosine deaminase acting on the RNA 1 gene is reported to encode a double-stranded RNA-specific adenosine deaminase, then can convert adenosine to inosine, disrupting the stability of double-stranded RNA (7). Inserting, deleting, converting, or modifying ribonucleotides are the essential processes of enzyme-catalyzed transformations. It is well-known that the adenosine deamination to inosine (A-to-I) is one of the most abundant RNA modifications and is catalyzed by ADAR in metazoans (8–10). If a mutated form of ADAR1 encounters amino acids, it cannot form an effective dimer structure and play its normal function, thus leading to the occurrence of disease (11). Previous studies have shown that defects in ADAR1 activity caused by AGS mutations could lead to elevated levels of cytoplasmic double-stranded RNA, and finally result in interferon induction. Normal ADAR1 activity could prevent these RNAs from binding to the cytoplasmic dsRNA sensor IFIH1 through producing multiple IU mismatches (10). ADAR is widely expressed in mammals and considered as one of the housekeeping genes, but the molecular pathogenesis of DSH has not been well-demonstrated until now. In addition to DSH, the first case of Aicardi-Goutières syndrome type 6 (AGS 6) caused by mutations of ADAR1 was reported in 2012, which mainly characterized by symptoms of neurological involvement, with most patients presenting with mental and motor retardation, irritability, dystonia, convulsions, microcephaly and cataract. Skin frostbite is the most common extraneurological symptom. So far, only four families with ADAR1 gene mutations have been reported in the world for co-morbidity of DSH and AGS.

Here, we present the first identified AGS 6 combined with DSH caused by a homozygous mutation of the ADAR1 gene in China, and review the previous literature.

## Case Presentation

**FIGURE 1:**
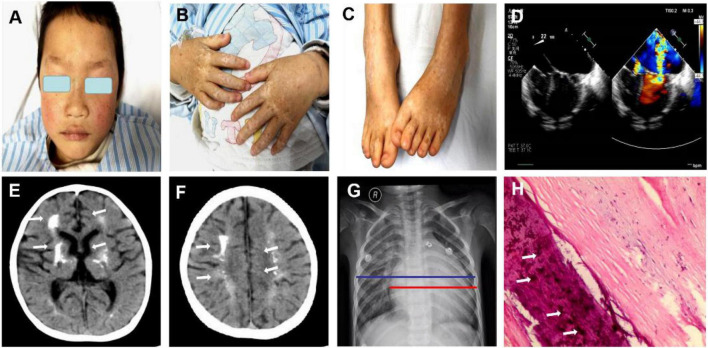
Clinical features of the patient with both dyschromatosis symmetrica hereditaria (DSH) and Aicardi-Goutières syndrome type 6 (AGS 6). The symmetrical appearance of freckle-like pigmentation and hypopigmentation spots were on the face **(A)**, backs of hands and feet **(B,C)**, presenting a reticulate pattern. Enlarged cardiac shadow was observed through Chest radiographs, and the cardio-thoracic ratio was around 0.65. **(D)** Cardiac color ultrasound showed normal postoperative cardiac changes after patent ductus arteriosus closure, severe mitral regurgitation (MR) and moderate mitral stenosis (MS), extremely mild aortic regurgitation (AR), mild + tricuspid regurgitation (TR) and a small amount of pericardial effusion (PE). **(E,F)** Brain computed tomography revealed calcification in the dentate nucleus, basal ganglia and cerebral white matter and mild brain atrophy. The textures of both lungs were increased and blurred, and there were small patchy shadows **(G)**. **(H)** A mitral valve biopsy revealed significant valve calcification, and the arrows marked the calcification.

**FIGURE 2:**
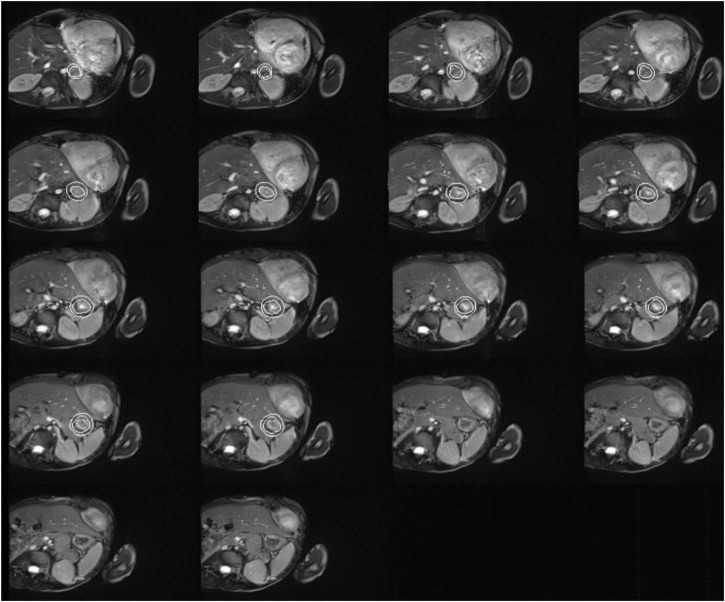
Cardiac magnetic resonance imaging of the patient with both dyschromatosis symmetrica hereditaria (DSH) and Aicardi-Goutières syndrome type 6 (AGS 6). Cardiac MRI image indicated mitral valve stenosis (MS), severe mitral regurgitation (MR), tricuspid regurgitation (TR), biatrial enlargement with the more significant left atrium, biventricular hypertrophy, and a small amount of pericardial effusion (PE).

A 5-year-old Chinese boy born to non-consanguineous parents presented with cardiac murmurs at birth and developmental retardation. His father was healthy, while his mother had miliary macules on both sides of the face. The patient experienced asphyxia neonatorum, with facial features of slightly ocular hypertelorism (Figures 1A–C) and a depressed nasal bridge. He could not raise his head at the age of 3 months followed by treatment with continuous exercise rehabilitation. In addition, he had miliary freckles on both cheeks and a high-arched palate at the age of 6 months. He could sit alone at 1 year of age, creep at 2 years old, and walk by age 5 but not steady. Until now, he could only utter simple syllables, not in phrases or sentences. When he was 3 years old, the patient received surgery to close the ductus under general anesthesia on August 10, 2017. Regrettably, he had a recurrent respiratory tract infection after the operation, improved with the treatment of cefotaxime sodium, furosemide, hydrochlorothiazide, spironolactone diuretic, and digoxin. On May 27, 2019, the child was admitted to the hospital after a severe cough and wheezing, edema of both eyelids, and decreased urine volume. On physical examination, there was mild edema of both eyelids, dry/moist rales could be heard in both lungs. Furthermore, a grade 4/6 systolic blowing murmur was audible in the precordial region with palpable tremors, and the murmur was carried extensively to the armpits. The liver was palpable at 4.5 cm below the costa. Multiple depigmentation spots of 0.5–1 cm in diameter were observed on the back of hands and feet. Moreover, he had bilateral wrist drop, slight varus and mild edema of both lower limbs, and normal muscle strength and muscle tension. Knee tendon reflex and achilles tendon reflex are normal, and pathological reflexes are negative. Laboratory examinations showed ALT 460.0 μ/l, AST 434.0 μ/l, BNP 4696.0 pg/ml, blood lactate 4.14 mmol/l, complement C3 0.45 g/l, complement C4 0.07 g/l. Enlarged cardiac shadow was observed through chest radiographs, and the cardio-thoracic ratio was around 0.65 (Figure 1G). The textures of both lungs were increased and blurred, and there were small patchy shadows. Calcification in the dentate nucleus, basal ganglia and cerebral white matter, and mild brain atrophy were revealed by brain computed tomography (Figures 1E,F). Cardiac color ultrasound showed normal postoperative cardiac changes after patent ductus arteriosus closure, severe mitral regurgitation (MR) and moderate mitral stenosis (MS), extremely mild aortic regurgitation (AR), mild + tricuspid regurgitation (TR), and a small amount of pericardial effusion (PE) (Figure 1D). Cardiac MRI image indicated MS, severe MR, TR, biatrial enlargement with the more significant left atrium, biventricular hypertrophy, and a small amount of PE (Figure 2). Subsequently, the patient was treated with cefotaxime sodium, digoxin for increasing myocardial contractility, hydrochlorothiazide, spironolactone for diuresis, and enalapril for promoting myocardial remodeling. However, he still had severe cough and cardiac insufficiency after effective treatment. On June 21, 2019, the ROSS-II procedure (mitral valve replacement with the pulmonary autograft) was performed under general anesthesia, and then mitral valve calcification was verified to result in severe mitral valve regurgitation (Figure 1H). Although the patient received symptomatic and supportive treatments after surgery, his cardiac function still got worsening, and massive mitral regurgitation was detected several months later, finally indicating a poor outcome.

## Exome Analysis

**FIGURE 3:**
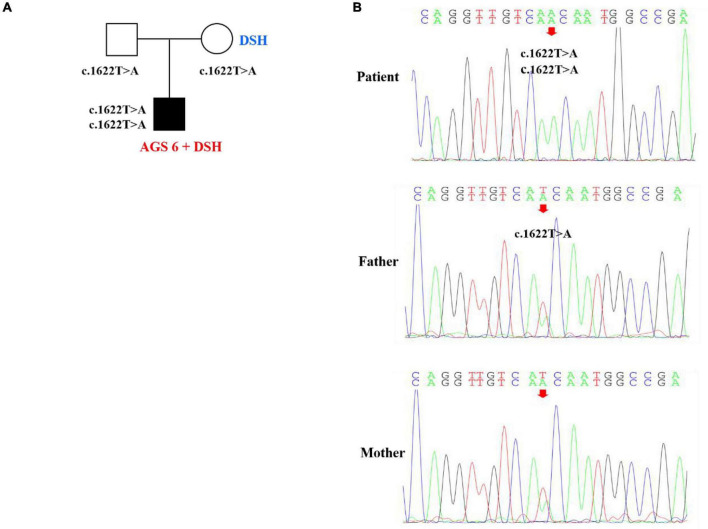
Pedigree features of the patient with both dyschromatosis symmetrica hereditaria (DSH) and Aicardi-Goutières syndrome type 6 (AGS 6) and the causative ADAR1 mutation. **(A)** Pedigree with the identified ADAR homozygous mutation. **(B)** Sequencing verification results showed that the homozygous nucleotide variation of ADAR gene c.1622T > A of the patient caused amino acid 541 to change from Ile to Asn, which was missense mutation, and was inherited from his parents, respectively.

Whole exome sequencing was performed with the consent of the proband’s parents (completed by Beijing Kangso Medical Laboratory), the suspected pathogenic loci were then verified by Sanger sequencing. The results showed that the homozygous nucleotide variation of ADAR1 gene c.1622T > A of the proband caused amino acid 541 to change from Ile to Asn, which was a missense mutation, and was inherited from his parents, respectively (Figure 3). The mutation was predicted damaging by SIFT and PolyPhen-2 and was predicted harmful using the ACMG criteria, and the locus was highly conserved among different species. The proband’s parents both carried heterozygous mutations of the same ADAR gene locus, and miliary depigmentation spots were also seen on the mother’s face, while the father was normal. Combined with the patient’s clinical phenotype, imaging characteristics, and exome sequencing results, the patient was diagnosed with AGS 6 and DSH.

## Literature Review

Patrizi et al. (12) reported a case of a 9-year-old white girl with DSH at the age of 2 and torsion dystonia at the age of 7, but did not describe whether the child had mental deterioration and brain calcification. Unfortunately, there were no pathogenic genes of DSH identified and no report on ADAR1 gene analysis at that time. Rice et al. collected data on 46 AGS patients from 37 families of panethnic origin with either biallelic mutations in ADAR1 (28 families) or the single known dominant-negative mutation p.Gly1007Arg (nine families), and also demonstrated 5 AGS patients had pigmentary lesions (13). To date, seven families of DSH combined with AGS 6 caused by compound heterozygous mutations of the ADAR1 gene have been reported. The phenotypic features were summarized in Table 1. Kono et al. reported a Japanese girl with an afebrile convulsive seizure at the age of 1 month, and neurological regression from 13 months, at 17 months old she could not sit unassisted regrettably (14). Intracranial calcification was detected when she was 4 months old, and worsened by 17 months. A mixture of hyper- and hypopigmented macules were developed on her whole body at the age of 5 months, as well as freckle-like macules on her face. Her mother also had slight skin lesions on her toes and fingers, but no nervous system involvement. Gene sequencing showed that the proband had a compound heterozygous mutation of the ADAR1 gene, i.e., c.1600C > T (p.Arg534X) (paternally derived) and c.3444-1G > A (IVS14-1G > A) (maternally derived). In 2018, another proband, an 11-year-old Chinese boy, was reported to experience developmental delay since born and visited the clinic for convulsions (15). He had symmetric mixed pigmentation and depigmentation spots on the backs of hands and feet, and freckle-like pigment spots on the face. Brain CT showed severe bilateral basal ganglia calcification, brain MRI suggested a thin corpus callosum, and extensive abnormal signals in both internal and external capsules and periventricular white matter and centrum semiovale white matter. The results of gene sequencing indicated that there was a compound heterozygous mutation in ADAR1 (c.1A > G and c.3124C > T). The proband’s elder brother also had the same genotype but only showed skin lesions clinically. Earlier in 2006 and 2008, the missense mutation (p.Gly1007Arg) in ADAR1 was reported to cause DSH with neurological symptoms in two families. One patient was an 11-year-old Japanese boy with major manifestations of skin pigmentation disorders, dystonia, developmental regression, and intracranial calcification (2). The other female patient, 27-year-old, was characterized by newborn skin pigmentation disorders, motor and mental regression, intracranial calcification, and cartilage calcification at the age of 17 (16). Psychic alterations and mental deterioration appeared when she was 21-year-old, including excitation and irritability. Her father also had depigmentation when he was young, and gradually lost some motor ability. After the age of 30, he experienced mental regression and severe aortic valve calcification. The missense mutation of c.3019G > A (p.G1007R) on exon 11 of the ADAR1 gene was found in both two families. Unfortunately, the genotype of the last proband’s father could not be detected due to his death, but both her mother and elder sister had no variants in ADAR1. Regrettably, it was only recognized that the two families were presented with organ calcification and neurological involvement on the basis of DSH at that time, unaware of the ADAR1 gene also possibly leading to AGS. Sathishkumar et al. reported three AGS 6 combined with DSH children in Indian. They were all characterized by global developmental delay and dystonia, along with dyschromatosis of the extremities and facial freckling. Exome analysis performed for two patients both revealed compound heterozygous variants in ADAR1, while the third patient did not perform the genetic analysis (17).

**TABLE 1:** Main clinical and genetics features of the patients with AGS 6 and DSH.

	Current paper		Kono et al. (14)		Xu et al. (15)		Kana et al. (23)			Sathishkumar et al. (17)		
						
	Patient	Mother	Patient	Mother	Patient	Brother	Patient	Father	Patient	P1	P2	P3
Mutations (cDNA)	c.1622T > A homozygous mutations	c.1622T > A heterozygous mutations	c.1600C > T c.3444-lG > A	C.1600C > T	c.1A > G c.3124C > T	c.1A > G c.3124C > T	c.3019G > A	Unknown	c.3019G > A	c.2271-3A > G c.578C > T	C.577C > G c.1314C > A	/
Protein	I541A		A534X		p.Met1? A1042C		G1007R		G1007R	VUS P193L	P193A Tyr438Ter	**/**
Origin	Chinese		Japanese		Chinese		Caucasian		Japanese	Indian	Indian	Indian
Gender	M	F	F	F	M	M	F	M	M	**/**	**/**	**/**
Epilepsy	No	No	Yes	No	Yes	No	No	No	No	No	No	No
Cognitive impairment	Yes	No	Yes	No	Yes	No	Yes	No	Yes	Yes	Yes	Yes
Dystonia	No	No	No	No	No	No	Yes	Yes	Yes	Yes	Yes	Yes
Skin pigmentation	Yes	Yes	Yes	Yes	Yes	Yes	Yes	Yes	Yes	Yes	Yes	Yes
White matter (MRI)	No	No	No	No	Yes	No	No	No	Yes	Yes	Yes	Yes
Calcification of heart valves	Yes	No	No	No	No	No	No	Yes	No	No	No	No
Intracranial calcinosis	Yes	No	Yes	No	Yes	No	Yes	No	Yes	No	Yes	Yes

Furthermore, ADAR mutations have been proved to enhance type I interferon signaling, perhaps inducing calcifying cardiac valve disease in children. Crow et al. (18) described three patients with biallelic ADAR mutations who developed calcifying cardiac valvular disease in late childhood. Exome sequencing showed the first patient and his younger sister both had compound heterozygous pathogenic ADAR variants c.577C > G; p.Pro193Ala (paternally derived) and c.3202 + 1G > A (maternally derived), and the last patient had other ADAR variants, c.518A > G; p.Asn173Ser and c.1552dup; p.Thr518Asnfs*31. Progressive calcification of the valvular leaflets was revealed by Echocardiography in all three patients, finally resulting in valvular stenosis. Despite maximum treatment, the disease progressed to severe heart failure causing death at age 17 in one case. Fortunately, another patient received mechanical mitral and aortic valve replacement when he was 16 years old and had a very good postoperative recovery.

## Discussion

In the present study, we reported the first patient identified AGS 6 combined with DSH resulting from the homozygous mutation of the ADAR1 gene (c.1622T > A), which was located in the coding sequence (ORF) and would lead to variation in protein structure. Notably, in addition to intracranial calcification and mitral valve calcification, the patient also experienced significant cardiac involvement, PDA, VSD, and progressive aggravated heart failure. Ultimately, we identified a novel ADAR1 variant and have added it to the phenotypic spectrum by providing details of the associated cardiac phenotype.

At present, there are more than 200 ADAR1 gene loci included in HGMD, among which 182 are DSH-related mutations. While only 15 are AGS-related mutations, and more than half of them are missense/non-sense mutations. Up to date, the mechanism of the ADAR1 gene mutations inducing DSH has been unclear, it might be associated with the counteraction of ADAR on the melanocyte apoptosis induced by stress. Noticeably, most DSH patients are East Asians (19), more than 20 cases of DSH caused by ADAR1 mutations have been reported in China, while only 1 Hispanic case of DSH has been reported in the Caucasian. This large racial difference could be attributed to racial skin color, the Caucasian has too lighter skin to recognize the loss of the pigment, while East Asians have darker skin to make the depigmentation more noticeable. The current patient showed classic freckle-like pigmentation and hypopigmentation spots on the hands and feet, which were inherited from his mother through the ADAR1 heterozygous mutation (c.1622T > A). It’s a surprise that the father also had the same mutation. Thus, our patient was diagnosed as suffering from AGS 6 and DSH with CHD and mitral valve calcification caused by an ADAR1 homozygous mutation.

In 2012, it was reported that ADAR1 mutations would cause a specific AGS phenotype, and also detected that the mutant genotype individuals had a high expression of type I interferon signaling similar to the ADAR1 null mouse *in vitro* (20). It is well-known that ADAR1 is an encoded dsRNA-specific adenosine dehydrogenase, and mediates RNA editing and protein synthesis in the nucleus. ADAR1 is widely expressed in CNS, especially the basal ganglia, and the reduced expression of ADAR1 could make neurons more susceptible to apoptosis after infection. It is reported that ADAR1 mutant mouse embryos had an aberrant interferon induction, and the embryonic lethality was rescued to live birth through supplement exogenous ADAR1. Besides, restoring the expression of cytoplasmic ADARs could alleviate aberrant immune responses in ADAR1 mutant mouse embryo fibroblasts *in vitro*. Mannion et al. (21) have verified that transfecting dsRNA oligonucleotides into ADAR1 mutant mouse embryo fibroblasts could alleviate the aberrant innate immune response *in vitro*. In AGS, ADAR1 mutations affected the activity of the interferon-inducible cytoplasmic isoform more severely than the nuclear isoform. In our study, the current patient also had a recurrent respiratory infection, which might be associated with the aberrant immune responses. Regrettably, we could not detect the type I IFN level in the patient’s cerebrospinal fluid due to the parents’ refusal. Further, ADAR1 also has proved to catalyze RNA editing at the Q/R sites of glutamate receptor subunits, then reduce the Ca^2+^ permeability of glutamate receptors in CNS (22). Mutations in ADAR1 could induce glutamatergic overactivity, and increase the toxic to neurons, finally inducing various neurological abnormalities, such as dystonia and mental deterioration by means of brain calcification (23).

## Conclusion

The current report further expands the phenotypic and genetic knowledge of ADAR1, in particular by reporting a novel pathogenic ADAR1 homozygous mutation and by describing progressive cardiac signs including congenital heart disease (CHD) and mitral valve calcification, which may be related to the mutation site located at the identified protein domain of ADAR1 gene. Therefore, more attention should be paid not only to the CNS and skin manifestations for children with a homozygous mutation of ADAR1, but also to heart and blood vessel involvement, so as not to aggravate the patient’s condition due to missed diagnosis.

## Data Availability Statement

The original contributions presented in the study are included in the article/supplementary material, further inquiries can be directed to the corresponding author/s.

## Ethics Statement

Written informed consent was obtained from the individual(s), and minor(s)’ legal guardian/next of kin, for the publication of any potentially identifiable images or data included in this article.

## Author Contributions

LJL, LQL, and DM: conception and design of the study. LZ and PH: performance of the experiments. LJL and CW: data analysis. LJL and JX: contributed reagents, materials, and analysis the tools. LJL and YX: the manuscript writing. All authors contributed to editorial changes in the manuscript and read and approved the final manuscript.

## Conflict of Interest

The authors declare that the research was conducted in the absence of any commercial or financial relationships that could be construed as a potential conflict of interest.

## Publisher’s Note

All claims expressed in this article are solely those of the authors and do not necessarily represent those of their affiliated organizations, or those of the publisher, the editors and the reviewers. Any product that may be evaluated in this article, or claim that may be made by its manufacturer, is not guaranteed or endorsed by the publisher.

## Abbreviations

DSH: dyschromatosis symmetrica hereditaria
ADAR1: adenosine deaminase acting on the RNA 1 gene
AGS 6: Aicardi-Goutières syndrome type 6
PDA: patent ductus arteriosus
VSD: ventricular septal defect
A-to -I: adenosine deamination to inosine
MR: mitral regurgitation
MS: moderate mitral stenosis
AR: aortic regurgitation
TR: tricuspid regurgitation
PE: pericardial effusion
BSN: bilateral striatal necrosis
CNS: central nervous system
CHD: congenital heart disease
PCR: polymerase chain reaction.

## References

[1] Suzuki N Suzuki T Inagaki K Ito S Kono M Horikawa T Ten novel mutations of the ADAR1 gene in Japanese patients with dyschromatosis symmetrica hereditaria. *J Invest Dermatol.* (2007) 127:309–11. 10.1038/sj.jid.5700528 16917490

[2] Kondo T Suzuki T Ito S Kono M Negoro T Tomita Y. Dyschromatosis symmetrica hereditaria associated with neurological disorders. *J Dermatol.* (2008) 35:662–6. 10.1111/j.1346-8138.2008.00540.x 19017046

[3] Kono M Akiyama M. Dyschromatosis symmetrica hereditaria and reticulate acropigmentation of Kitamura: an update. *J Dermatol Sci.* (2019) 93:75–81. 10.1016/j.jdermsci.2019.01.004 30692041

[4] Kondo T Suzuki T Mitsuhashi Y Ito S Kono M Komine M Six novel mutations of the ADAR1 gene in patients with dyschromatosis symmetrica hereditaria: histological observation and comparison of genotypes and clinical phenotypes. *J Dermatol.* (2008) 35:395–406. 10.1111/j.1346-8138.2008.00493.x 18705826

[5] Pestal K Funk CC Snyder JM Price ND Treuting PM Stetson DB. Isoforms of RNA-editing enzyme ADAR1 Independently control nucleic acid sensor MDA5-driven autoimmunity and multi-organ development. *Immunity.* (2015) 43:933–44. 10.1016/j.immuni.2015.11.001 26588779 PMC4654992

[6] Guo X Liu S Yan R Nguyen V Zenati M Billiar TR ADAR1 RNA editing regulates endothelial cell functions via the MDA-5 RNA sensing signaling pathway. *Life Sci Alliance.* (2022) 5:e202101191. 10.26508/lsa.202101191 34969816 PMC8739526

[7] Keegan LP Vukić D O’Connell MA. ADAR1 entraps sinister cellular dsRNAs, thresholding antiviral responses. *Trends Immunol.* (2021) 42:953–5. 10.1016/j.it.2021.09.013 34642093

[8] Nakahama T Kato Y Shibuya T Inoue M Kim JI Vongpipatana T Mutations in the adenosine deaminase ADAR1 that prevent endogenous Z-RNA binding induce Aicardi-Goutières-syndrome-like encephalopathy. *Immunity.* (2021) 54:1976–88.e7. 10.1016/j.immuni.2021.08.022 34525338

[9] Song B Shiromoto Y Minakuchi M Nishikura K. The role of RNA editing enzyme ADAR1 in human disease. *Wiley Interdiscip Rev RNA.* (2021) 13:e1665. 10.1002/wrna.1665 34105255 PMC8651834

[10] Guo X Wiley CA Steinman RA Sheng Y Ji B Wang J Aicardi-Goutières syndrome-associated mutation at ADAR1 gene locus activates innate immune response in mouse brain. *J Neuroinflammation.* (2021) 18:169. 10.1186/s12974-021-02217-9 34332594 PMC8325854

[11] Chung H Calis JJA Wu X Sun T Yu Y Sarbanes SL Human ADAR1 prevents endogenous RNA from triggering translational shutdown. *Cell.* (2018) 172:811–24.e14. 10.1016/j.cell.2017.12.038 29395325 PMC5831367

[12] Patrizi A Di Lernia V Neri I Badiali De Giorgi L Masi M. Epidermolysis bullosa simplex associated with muscular dystrophy: a new case. *Pediatr Dermatol.* (1994) 11:342–5. 10.1111/j.1525-1470.1994.tb00102.x 7899187

[13] Rice GI Kitabayashi N Barth M Briggs TA Burton ACE Carpanelli ML Genetic, phenotypic, and interferon biomarker status in ADAR1-related neurological disease. *Neuropediatrics.* (2017) 48:166–84. 10.1055/s-0037-1601449 28561207 PMC5985975

[14] Kono M Matsumoto F Suzuki Y Suganuma M Saitsu H Ito Y Dyschromatosis symmetrica hereditaria and Aicardi-Goutières syndrome 6 are phenotypic variants caused by ADAR1 mutations. *J Invest Dermatol.* (2016) 136:875–8. 10.1016/j.jid.2015.12.034 26802932

[15] Xu M Guo H Lu XP. Clinical, pedigree and genetic analysis of Aicardi-Goutières syndrome type 6 in a patient. *J Clin Pediatr.* (2018) 36: 686–8.

[16] Tojo K Sekijima Y Suzuki T Suzuki N Tomita Y Yoshida K Dystonia, mental deterioration, and dyschromatosis symmetrica hereditaria in a family with ADAR1 mutation. *Mov Disord.* (2006) 21:1510–3. 10.1002/mds.21011 16817193

[17] Sathishkumar D Muthusamy K Gupta A Malhotra M Thomas M Koshy B Co-occurrence of Case Report: Aicardi-Goutières Syndrome Type 6 and Dyschromatosis symmetrica hereditaria due to compound heterozygous pathogenic variants in ADAR1: a case series from India. *Clin Exp Dermatol.* (2021) 46:704–9. 10.1111/ced.14531 33289110

[18] Crow Y Keshavan N Barbet JP Bercu G Bondet V Boussard C Cardiac valve involvement in ADAR -related type I interferonopathy. *J Med Genet.* (2020) 57:1–4. 10.1136/jmedgenet-2019-106457 31772029

[19] Vaughn MG Salas-Wright CP DeLisi M Larson M. Deliberate self-harm and the nexus of violence, victimization, and mental health problems in the United States. *Psychiatry Res.* (2015) 225:588–95. 10.1016/j.psychres.2014.11.041 25500323

[20] Rice GI Kasher PR Forte GM Mannion NM Greenwood SM Szynkiewicz M Mutations in ADAR1 cause Aicardi-Goutières syndrome associated with a type I interferon signature. *Nat Genet.* (2012) 44:1243–8. 10.1038/ng.2414 23001123 PMC4154508

[21] Mannion NM Greenwood SM Young R Cox S Brindle J Read D The RNA-editing enzyme ADAR1 controls innate immune responses to RNA. *Cell Rep.* (2014) 9:1482–94. 10.1016/j.celrep.2014.10.041 25456137 PMC4542304

[22] Wang Q Khillan J Gadue P Nishikura K. Requirement of the RNA editing deaminase ADAR1 gene for embryonic erythropoiesis. *Science.* (2000) 290:1765–8. 10.1126/science.290.5497.1765 11099415

[23] Tojo K Sekijima Y Suzuki T Suzuki N Tomita Y Yoshida K Dystonia, mental deterioration, and dyschromatosis symmetrica hereditaria in a family with ADAR1 mutation. *Mov Disord*. (2006) 21:1510–13. 10.1002/mds.21011 16817193

